# Polymyxin B hemoadsorption in endotoxic septic shock: ten questions on the Tigris trial

**DOI:** 10.1186/s13054-026-06219-y

**Published:** 2026-08-27

**Authors:** Matthieu Legrand, Javier Neyra, George Tomlinson, John A Kellum

**Affiliations:** 1 Department of Anesthesia and Perioperative Care, Division of Critical Care Medicine, University of California, 521 Parnassus Avenue, San Francisco, CA 94143 USA; 2 Department of Medicine, Division of Nephrology, University of Alabama at Birmingham, Birmingham, AL USA; 3 Dalla Lana School of Public Health, University of Toronto, Toronto, ON Canada; 4 Department of Critical Care Medicine, University of Pittsburgh, Pittsburgh, PA USA; 5 Spectral Medical Inc, Toronto, ON Canada

**Keywords:** Septic shock, Endotoxin, Polymyxin B, Hemoadsorption, Bayesian trial, Precision medicine, Extracorporeal therapy, Hemoperfusion

## Abstract

The Tigris trial recently reported that polymyxin B hemoadsorption (PMX) achieved a high posterior probability of benefit for mortality in adults with endotoxic septic shock (endotoxemia with multiple organ failure). The trial used a Bayesian framework with a prior derived from a subgroup of an earlier trial (EUPHRATES) and this subgroup was used to design eligibility criteria for Tigris. Ten questions that expose the trial's strengths and limitations are answered in this review. Writing as the trial investigators, our intention is to make the reasoning behind these decisions explicit, so that clinicians and trialists can judge what Tigris does, and does not, demonstrate.

## Background

Septic shock remains a leading cause of death in the intensive care unit, and despite three decades of randomized trials only one specific pharmacological therapy was shown to modify its trajectory once organ failure is established. This therapy, drotrecogin alfa, was initially approved but was later withdrawn as its safety and efficacy could not be confirmed in subsequent trials [1]. Endotoxin, the lipopolysaccharide of Gram-negative cell walls, is a biologically plausible target: a proximal driver of innate immune activation whose activity can be quantified at the bedside by the endotoxin activity assay (EAA). Polymyxin B hemoadsorption (PMX, Toraymyxin) selectively binds endotoxin via polymyxin B immobilized on a polystyrene-derivative fiber [2, 3]. Three previous randomized trials—EUPHAS [4], ABDO-MIX [5] and EUPHRATES [6]—produced discordant results. Post-hoc analysis of EUPHRATES identified a biologically coherent subgroup defined by intermediate endotoxin activity (EAA 0.60–0.89) and severe organ dysfunction in whom PMX appeared to confer survival benefit, reflecting both predictive enrichment (presence of the therapeutic target) and prognostic enrichment (sufficient event rate—mortality) [7].

**Fig. 1:**
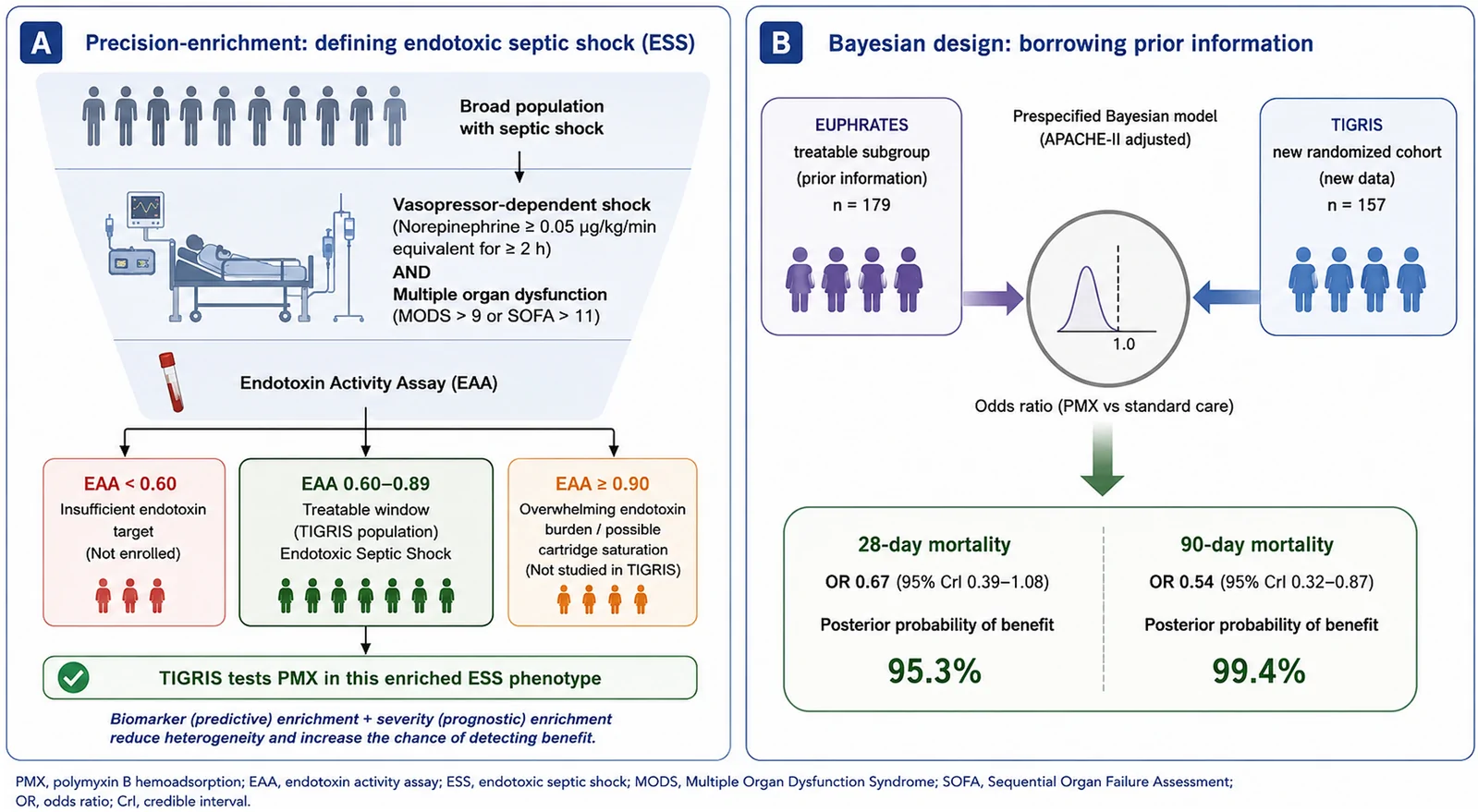
Precision-enrichment strategy and Bayesian design of the TIGRIS trial. Panel **A**: enrichment strategy of the Tigris trial. Panel **B**: prespecified APACHE-II–adjusted Bayesian model combining prior information from the EUPHRATES treatable subgroup with the new randomized TIGRIS cohor

The Tigris trial was designed to test this hypothesis prospectively in a Bayesian phase 3 framework, borrowing data from the treatable subgroup in EUPHRATES to increase power while requiring confirmation in an independent new population [8] (Fig. 1). Published results indicate a posterior probability of benefit of 95.3% at 28 days (APACHE-II adjusted odds ratio 0.67, 95% credible interval 0.39–1.08) and 99.4% at 90 days (odds ratio 0.54, 95% credible interval 0.32–0.87), with absolute risk reductions of approximately 10% and 15%.

In this manuscript, we discuss the methodology and results of the Tigris trial in the form of questions & answers. We write as investigators of the Tigris trial, so this commentary is our own account of the trial’s rationale, design, and interpretation rather than an independent appraisal; we set out the reasoning transparently, including where the evidence is most open to challenge. These questions emerged from the peer-review exchange preceding publication and from recurrent questions raised by clinicians and trialists.

**Question 1. Tigris was an open-label trial. How can we be sure the results are not driven by differential co-interventions or biased outcome ascertainment?**

***Response.*** Blinding an extracorporeal therapy requiring vascular access, a circuit and two cartridge sessions is not feasible without a sham device, which raises its own objections. Both EUPHRATES and Tigris trials mitigated bias in two ways. First, site investigative teams were operationally separated from the clinical teams ordering standard therapies. Second, the primary endpoint—mortality at 28 and 90 days—is objective and vital-status–ascertained, leaving no scope for adjudication bias. Although vital status is not susceptible to ascertainment bias, open-label allocation can still influence outcome through co-interventions—organ-support escalation or de-escalation, renal-replacement timing, ventilatory and vasopressor intensity, ICU discharge timing, and decisions to limit life-sustaining therapy. We therefore do not claim the open-label design could not influence outcomes, only that the objective mortality endpoint removes one important pathway of bias. There is, moreover, no evidence that any such differential co-intervention occurred: for example, more control-arm than PMX-arm patients remained on life support in the ICU at day 28 (21.4% vs 13.8%), so any residual effect of the open-label design would have biased the result toward the null [8].

**Question 2. Of nearly 15,000 patients screened, only 157 were enrolled. Doesn't a 1% inclusion rate undermine generalizability?**

***Response.*** The 1% headline figure is misleading, reflecting trial logistics as much as patient characteristics. Of 14,890 screened, only 328 were clinically eligible; most were excluded not for endotoxin status but for not meeting the severity criteria (vasopressor-dependent septic shock, MODS >9 or SOFA >11). Of the 328 with EAA measured, 87 were below 0.60, 42 ≥0.90, and 5 EAA non-responders, leaving 194 in the 0.60–0.89 window, of whom 157 were randomized. Eden, a parallel study at four Tigris sites, found 21% of septic-shock patients met full criteria for endotoxic septic shock (ESS). The contrast between this 21% and the ~2% randomized reflects different denominators: the Tigris denominator was all screened patients (most excluded for severity criteria or never EAA-tested), whereas Eden applied the same criteria to an established-septic-shock cohort, giving prevalence among the at-risk population. Thus, ESS is uncommon but not rare, and the low randomization fraction reflects patient selection and trial logistics more than the true population prevalence. We acknowledge, nonetheless, that the stringent eligibility criteria—while sharpening the “high EAA and organ failure” phenotype and strengthening internal validity—necessarily limit the generalizability of the findings to all sepsis comers and confine the evidence base to a precisely defined subset of patients with septic shock. The implication is straightforward: Tigris does not license PMX in unselected septic shock, only in patients meeting the prospectively defined “high EAA and organ failure” phenotype [9]. Patients with EAA values of 0.90 or above (13% of patients screened for Tigris), in whom endotoxin burden is highest and dose-adjusted strategies might be more effective, were not studied and remain an important target for future investigation.

**Question 3. How does the Bayesian inference in Tigris differ from a frequentist analysis, and how many patients would a frequentist trial have required to reach the same conclusion?**

***Response.*** These are, in our view, the most consequential methodological questions that Tigris raises. In simplest terms, a frequentist analysis asks: under the assumption that the treatment has no effect, what is the probability of observing a trial result at least as extreme as the observed result? It answers a question about other potential trial results. By contrast, a Bayesian analysis asks: when we combine prior information about a treatment effect with these observed trial data, what is the resulting probability that the treatment is beneficial? Both frameworks are legitimate, but they answer different questions; only the Bayesian framework can deliver a direct probability statement of the form "there is a 95.3% probability that PMX is beneficial". Three concrete comparisons illustrate this difference. First, a frequentist design of the Tigris trial would have started from scratch, with no borrowing of information from EUPHRATES. The results of such a trial could be pooled with EUPHRATES later in a meta-analysis, but the frequentist Tigris would have needed to be designed with adequate power to stand alone. The sample size needed for a trial to detect a mortality reduction of 10% from a control group mortality of 45% is n=592 assuming a one-sided alpha of 0.05 and 80% power. Thus, by prespecifying an informative prior anchored to the only previous trial that had studied the same patient population, Tigris reached an interpretable answer with 157 new patients—roughly 4-times fewer than a conventional design would have required. This is the practical case for Bayesian methods when the eligible patient is rare: frequentist sample sizes are often prohibitive, and structured prior borrowing offers a feasible route to a definitive answer [10, 11]. If EUPHRATES had merely stopped enrolling patients with EAA ≥ 0.90 and continued enrolling patients with EAA in the 0.60 to 0.89 range with MODS > 9 (an enrichment phase), it would resemble large platform trials that have had so much success recently [12, 13]. If so, the treatment effect could have been estimated from all EAA-defined-subgroup patients across both the pre- and post-enrichment phases. Pooling in this way is equivalent to forming a prior from the first part of the trial, putting 100% weight on it (Tigris discounted this by 25%), and updating with the second part. Finally, a Bayesian analysis without an informative prior still gives a posterior probability of benefit of ~80% at 28 days and above 98% at 90 days—showing the 90-day result does not depend on borrowing from the prior.

**Question 4. The primary odds ratios are adjusted for APACHE-II. Why adjust at all in a randomized trial, and does the adjustment, rather than the treatment, drive the result?**

***Response.*** Adjustment for a strong prognostic covariate in a randomized trial is not a correction for post-randomization imbalance but a prespecified strategy to improve power. The primary analysis adjusted the treatment effect for baseline APACHE-II, strongly associated with mortality in this population. In the primary logistic regression model, conditioning on APACHE-II yields an estimate closer to the clinically meaningful conditional effect at the bedside. A Bayesian analysis that borrows external information through an informative prior, with a prespecified covariate adjustment, is precisely the kind of efficient design suggested by guidance in three separate FDA documents [14–16]. The last two address the use of Bayesian methods to support primary inference in pivotal trials and explicitly contemplate borrowing information from previously available trials, while emphasizing that the prior distribution, success criteria, analysis model and operating characteristics be specified in advance. Two features of Tigris make the concern that adjustment *created* the result untenable. First, the adjustment variable and the model were fixed in the statistical analysis plan before the trial started, so the analysis is not a post-hoc search for a covariate that produces a favorable answer. Second, unadjusted and alternatively adjusted analyses give qualitatively concordant estimates compared to the adjusted analysis. Adjustment provides something closer to a patient-specific treatment effect; it does not manufacture one. The implication for readers is that the prespecified adjusted analysis is a more, not less, trustworthy estimation of the treatment effect.

**Question 5. The Bayesian primary analysis used a 75% weight on the EUPHRATES-derived prior. Why 75% rather than say 50% or 100%, and how sensitive are the conclusions to that choice?**

***Response.*** All informative priors involve a judgement that should be transparent. The 75% weight was prespecified and deliberately down-weights the EUPHRATES treatable-cohort information, giving an effective prior sample size (~134) smaller than the actual cohort (n=179). One useful way to read the design is by analogy to adaptive trials: the EUPHRATES treatable cohort plays the role of an interim analysis, and Tigris the role of the confirmatory continuation [7, 8]. The analogy is imperfect, however: in a genuine adaptive enrichment trial the enrichment rule is fixed prospectively, whereas here it was learned from the first dataset and the prior population identified post-hoc, so we offer it as an aid to intuition rather than a claim of equivalence. This distinction is implicitly acknowledged in the down-weighting: had Tigris been a true adaptive trial, the subgroup showing benefit could have been retained without discounting. This holds because the two studies share eligibility criteria (vasopressor-dependent septic shock, MODS >9 or SOFA >11, EAA 0.60–0.89), the only difference being that the EAA range was defined post-hoc in EUPHRATES and prespecified in Tigris—which the 25% discount reflects. Because Tigris proceeded on a positive subgroup signal, that signal is subject to the “winner’s curse,” whereby an effect selected for being positive tends to overestimate the true benefit. Combining such a prior with unbiased Tigris data is precisely what the down-weighting is meant to mitigate. The down-weighting addresses this by reducing the prior’s effective influence. We do not claim 25% is formally optimal; it was a deliberate, prespecified judgement, defensible on three grounds: it was fixed before the trial comensed, the conclusions are reported across the full 0–100% weight range, and prior-data conflict diagnostics found no inconsistency. Reassuringly, Tigris 28-day mortality was close to the EUPHRATES treatable-subgroup rate, supporting the consistency of the effect size. For 28-day mortality, the posterior probability of benefit adjusted by APACHE-II exceeds 95% for any weight of 72% or greater, reaching 97.1% at full weight. For 90-day mortality, the result is essentially invariant: posterior probability of benefit remains above 98% even with an uninformative prior, meaning that the 90-day signal is mostly driven by the Tigris data. In short, the 75% weighting is reasonable and prespecified; the strength of the 28-day inference depends on the weight, whereas the 90-day inference does not.

**Question 6. At 28 days the credible interval for the odds ratio includes 1.0 (0.39–1.08), yet the posterior probability of benefit is 95.3%. How can both be true?**

**Fig. 2:**
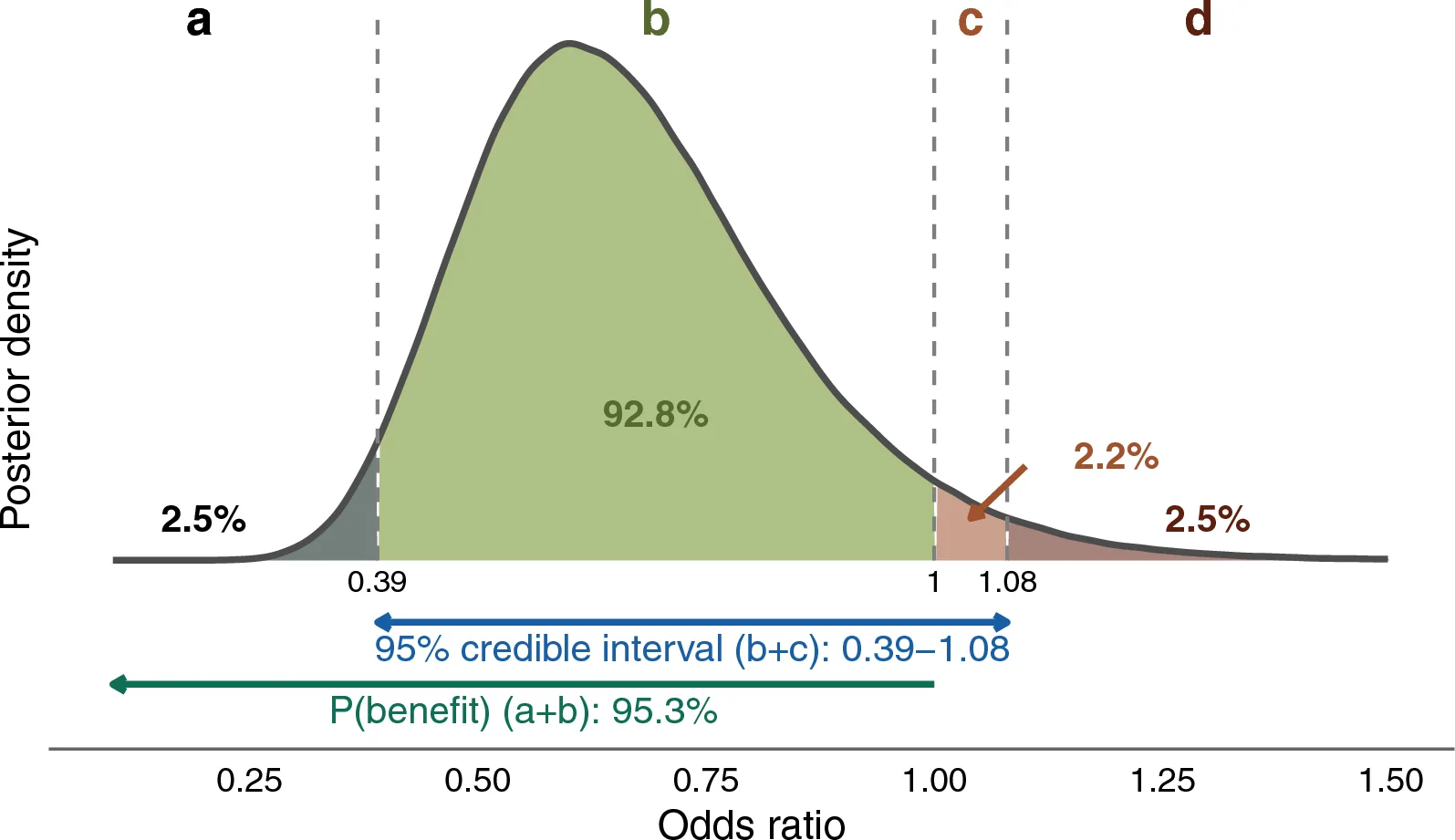
Posterior distribution of the odds ratio for mortality (PMX vs standard care). The posterior is divided into four portions for the OR: (**a**) P(OR < 0.39) = 2.5%; (**b**) P(0.39 < OR < 1.00) = 92.8%; (**c**) P( 1.00 < OR < 1.08) = 2.2%; (**d**) P(OR > 1.08) = 2.5% (figure 2). A two-sided 95% credible interval (**b**+**c**) can therefore include 1.0 even though the probability of benefit (**a**+**b**) is > 95%. This happens whenever the one-sided posterior probability of benefit lies between 95.0% and 97.5%

***Response.*** This apparent inconsistency can lead to confusion in Bayesian trial reporting. Based on the numbers above, we can assign posterior probabilities to these four ranges for the OR: (a) P(OR < 0.39) = 2.5%; (b) P(0.39 < OR < 1.00) = 92.8%; (c) P( 1.00 < OR < 1.08) = 2.2%; (d) P(OR > 1.08) = 2.5% (Fig. 2) [17, 18]. A two-sided 95% credible interval (b+c) can therefore include 1.0 even though the probability of benefit (a+b) is > 95%. This happens whenever the one-sided posterior probability of benefit lies between 95.0% and 97.5% as it did for 28-day mortality. In Tigris at 28 days, only 4.7% (c+d) of the posterior distribution lies above an odds ratio of 1.0, so the cumulative probability of benefit is 95.3%, while the upper bound of the equal-tailed interval stretches just past 1.0 to 1.08. This is the analog of a one-sided test reaching p<0.05 while a two-sided test does not; the clinically relevant question—whether PMX is better than standard of care—is one-sided, and the trial answers it. Prespecified error-rate analyses in the appendix were reassuring, with <1% probability of wrongly concluding benefit at 28 days.

**Question 7. The mortality difference between arms widened from day 28 to day 90. Is that biologically plausible, or a chance finding?**

***Response.*** Widening of a treatment effect over time is not uncommon in critical-illness trials. Two observations are relevant. First, organ-failure resolution—not hemodynamic stabilization—appears to be the dominant mechanism. Of patients alive at day 28, more control- than PMX-arm survivors remained in the ICU (21.4% vs 13.8%); of those, no control patient was discharged before day 90 (5 of 6 died), versus over half the PMX patients (6 of 9 survived). ICU-free survival at day 28 was 52.8% vs 43.1%. These signals suggest that, beyond mortality, the benefit of PMX operates through restoration of organ function—greater liberation from ventilation and organ support, earlier discharge among survivors—rather than acute hemodynamic change. As with most trials, however, Tigris was designed to estimate the probability of benefit rather than its mechanism, which future research must address. Second, most deaths beyond the first ICU week follow limitation of life-sustaining therapy in patients whose organ failure has not resolved, so a mortality difference between day 28 and day 90 reflects a difference in the proportion recovering enough to be liberated from organ support. The pattern of differential mortality effects persisting for 90 days following recruitment is also consistent with other recent ICU trials in which short-term differences mature into more pronounced long-term differences once critical illness resolves [19]. It could be suggested that, because the trial was open-label, the widening difference could reflect differential care decisions rather than biology. But non-resolution of organ dysfunction and the decision to limit life-sustaining therapy are not in tension: care-limitation is not arbitrary but follows non-resolution, so differential mortality and differential care-limitation are two expressions of the same difference in organ-failure resolution. We nonetheless present this mechanism as hypothesis-generating.

**Question 8. If endotoxin causes shock, why didn’t the intervention have a significant effect on hemodynamics?**

***Response.*** This finding departs from a long-standing assumption. Earlier studies framed PMX as rescue therapy for refractory shock, expecting endotoxin removal to raise blood pressure and cut vasopressor dose. In Tigris, mean arterial pressure and norepinephrine equivalents did not differ meaningfully over the first three days, yet 28- and 90-day mortality were lower with PMX. This dissociation is unsurprising: a consistent message from the past decade of septic-shock trials is that, varying the intensity of resuscitation does not improve survival. The CLOVERS trial [20], comparing a vasopressor-liberal with a fluid-liberal strategy, and the ARISE FLUIDS trial, comparing restrictive with liberal fluid resuscitation, both found no difference in mortality [21]. Together with fluid-strategy and early goal-directed therapy trials, these data argue that arterial pressure in septic shock is largely a marker of severity rather than a modifiable mediator of outcome. Endotoxin, by contrast, sits upstream of pathways of organ injury that resolve over weeks, not hours. Hemodynamic trajectory was only assessed on Days 0–3 in survivors, so hemodynamic endpoints are less relevant for judging endotoxin-directed therapy.

**Question 9. How was the PMX procedure delivered, how were the dose and anticoagulation chosen, and what was done in patients already receiving renal replacement therapy?**

***Response.*** The treatment was standardized to match exactly the intervention that generated the EUPHRATES treatable-cohort signal, a precondition for the Bayesian borrowing in Question 3. Each patient received two 2-hour hemoperfusion sessions about 22–24 hours apart, blood perfused at 80–100 mL/min through a double-lumen catheter. The fixed two-session course reflects cartridge adsorption capacity, which approaches saturation within a single 2-hour session, so adsorbed endotoxin per session is bounded (about 10–20 µg) [9]. The circuit was anticoagulated with unfractionated heparin, consistent across EUPHRATES and Tigris. In patients already on continuous renal replacement therapy (CRRT), CRRT was interrupted for the two hours of each PMX session and resumed afterward. The Tigris result therefore applies to this specific, reproducible procedure—two fixed 2-hour sessions roughly a day apart at 80–100 mL/min, heparinized, with any concurrent CRRT interrupted for the session. Translating this into routine practice carries a non-trivial resource burden; PMX is most readily deliverable in centres already equipped for extracorporeal therapies with timely EAA measurement. Patients with EAA ≥0.90 may have an endotoxin burden exceeding the capacity of a two-session course, so the standardized schedule may be insufficient; intensified regimens (additional sessions or shorter intervals) might be required, but were not explored in Tigris, as treatment had to align with EUPHRATES.

**Question 10. How does Tigris reconcile with prior negative trials and meta-analyses of polymyxin B hemoadsorption?**

***Response.*** PMX has been studied for more than three decades, and the trial record is, on its surface, contradictory. EUPHAS reported improved hemodynamics and survival in 64 patients with abdominal septic shock but did not use EAA-based selection and was stopped early [4]. ABDO-MIX, with 243 patients with peritonitis-associated septic shock, was neutral; it also did not use EAA selection, and approximately one third of patients did not complete the planned two cartridge runs [5]. Severity of patients was much lower than EUPHAS (or Tigris) with a control group mortality below 20%. EUPHRATES, the largest randomized PMX study with 450 patients, was overall negative for its primary endpoint [6]. A subsequent network meta-analysis judged the totality of evidence to be of low certainty [22]. The reconciling concept is that no earlier trial, except partly EUPHRATES, prospectively defined the subgroup in whom endotoxin removal could plausibly work. EUPHAS and ABDO-MIX did not measure endotoxin activity, wrongly assuming abdominal sepsis was a sufficient surrogate. EUPHRATES required EAA ≥0.60 but 17% had EAA ≥0.90 and did not restrict organ-failure severity. The post-hoc treatable cohort (EAA 0.60–0.89, MODS >9) is exactly what Tigris prespecified and confirmed. Patients with EAA ≥0.90 did not benefit, plausibly because the column saturates and this group has an overwhelming endotoxin burden—greater than 4000 pg/ml (> 20μg in an average adult), possibly much greater and beyond the capacity of the PMX cartridge to adsorb (~10-20μg of endotoxin in 2 hours) [9]. The pattern across trials is therefore not contradictory but informative: when the population is unselected or includes patients outside the treatable range, average effect is attenuated, sometimes approaching the null; when restricted (enriched) to the treatable endotype, a benefit emerges—the same logic that has converted indiscriminate biological-therapy trials into positive results in oncology once biomarker enrichment isolates the responsive subgroup. Importantly, the discovery of the precise signaling pathways for endotoxin were mapped only after PMX was developed. Clinical phenotyping alone is inadequate for selection since ESS and non-ESS share clinical features. Three implications follow. First, EAA testing should be considered in septic shock with multiorgan failure being assessed for adjunctive extracorporeal therapy. Second, PMX should be used only for ESS—EAA 0.60–0.89 with vasopressor-dependent septic shock and multiple organ failure—not extrapolated to lower-EAA or lower-severity patients. Patients with EAA ≥0.90 did not benefit under the standardized protocol, so the Tigris findings do not apply to them and further research is needed. Clinicians should read Tigris against the Surviving Sepsis Campaign guidelines [23], which do not at present recommend blood purification or endotoxin-removal therapies for routine use in sepsis. These recommendations, were formulated before the Tigris trial was reported and therefore do not yet incorporate its findings. Tigris does not overturn that position for unselected patients; rather, it identifies a narrow, biomarker-defined endotype—vasopressor-dependent septic shock with multiple organ failure and EAA 0.60–0.89—in which adjunctive PMX may be considered.

## Conclusions

Tigris provides the most methodologically rigorous evidence to date that endotoxin removal with polymyxin B hemoadsorption reduces mortality in a precisely defined subgroup of patients with ESS. Residual uncertainty remains, principally around the mechanism of benefit—most plausibly resolution of organ failure—and around the management of patients who fall outside the strict inclusion criteria. Tigris also illustrates that precision medicine is becoming methodologically tractable in critical care: biomarker-anchored, prognostically enriched eligibility combined with Bayesian priors can together provide an answer to difficult questions even in a small critically ill population. Whether the field is willing to adopt this template more broadly is a question the next generation of sepsis trials will have to confront.

## Data Availability

No datasets were generated or analysed during the current study.
